# Address on Spirituous Liquors

**Published:** 1828-10

**Authors:** 


19. *Dr. Mussey on Intemperance.* It is encouraging to observe so many societies directing their attention to the means of correcting the abuse of spirituous liquors. That they are the causes—immediate or remote—of a multitude of diseases, both of body and mind, no physician of observation can doubt. They clearly fall, therefore, within the legitimate scope of the profession; and should attract more of its attention than they have heretofore done.

On the 5th of June 1827, Dr. R. D. Mussey, professor of Anatomy and Surgery in Dartmouth College, and at that time President of the New-Hampshire Medical Society, delivered before the latter institution an "Address on Ardent Spirit," which at the request of the Society, was published within the present year.

As this address—respectable of its kind—is in a great measure declamatory, we shall not undertake its analysis, but the following extract which contains the professor's views on the *modus operandi* of ardent spirits, and involves some disputed points in physiology, will not be read without interest.

"What is the secret of this witchery which strong drink exerts over the whole man? I will try to tell you. After being received into the stomach it is sucked up by absorbent vessels, is carried into the blood, and circulates through the alimentary organs, through the lungs, muscles, and brain, and doubtless through every organ of the body. Not a blood vessel however minute, not a thread of nerve in the whole animal machine escapes its influence. What is the nature of this influence? It disturbs the functions of life; it increases for a time, the action of living organs, but lessens the power of that action; hence the deep depression and collapse which follow preternatural excitement. By habitual use, it renders the living fibre less and less susceptible to the healthy operation of unstimulating food and drink, its exciting influences soon become incorporated with all the living actions of the body, and the diurnal sensations of hunger, thirst, and exhaustion, are strongly associated with the recollection of its exhilarating effects, and thus bring along with them, the resistless desire for its repetition.

Is evidence required of its being absorbed, and pervading the different organs of the body? Approach within a few feet of the rum or brandy drinker, and the odour of his breath will quickly demonstrate, that the lungs, loaded with the foul liquor, are discharging it with all the energy in their power.

When taken by the nursing mother, it enters into the delicate food prepared by nature for the nourishment and growth of helpless infancy, and in this way, as may most rationally be supposed, produces a relish for an article naturally disgusting, and lays thus early, in some instances, a foundation for intemperance in after life. What physician has not known a nursing mother give a fretful child a good night's sleep, by taking, herself, a dose of brandy at bed time?

Other organs than those destined for the formation of milk, manifest the presence of this article when it is combined with peculiar odours; those organs especially, which are set as waste gates to the system, soon show how foreign it is, and ill adapted to the real wants of the animal economy, by separating it from the blood and taking it out of the general circulation as fast as possible.

The brain, that most delicate and wonderful organ, which forms the mysterious link between the other forms of matter and mind, the healthy functions of which are essential to vigorous intellectual operation, is capable of imbibing alkohol, and having all its actions suddenly arrested. In point, is the case of the man who was picked up dead in London, soon after having drank a quart of gin upon a wager. He was carried into the Westminster hospital and there dissected. "In the ventricles of the brain was found a considerable quantity of limpid fluid, distinctly impregnated with gin, both to the sense of smell and taste, and even to the test of inflammability. The liquid appeared, to the senses of the examining students, as strong as one third gin to two thirds water."

We know that alkohol, even when diluted, by long contact after death, hardens the brain, as well as the other soft textures of the body which contain albumen; and although the vital principle may enable the brain to resist in a great measure, and for a long time, this effect of alkohol, when brought into it from the stomach by the general circulation, the fact, as alleged by many, and as I am strongly induced to believe from the limited means I have had of observing, viz. that the brains of drunkards are literally harder at death, than those of the temperate, may be considered in strict accordance with the effects of intemperance upon the intellectual functions. If this organ be in any degree hardened by the circulation of diluted alkohol through its minute and most delicately organized parts, it might well be supposed to be less susceptible of those exquisitely balanced actions, which we can hardly help believing do exist in the impressions made by external objects, and in the variety of combinations of them, produced by the more abstract, and retired operations of the mind. That a large proportion of tiplers early discover an unnatural obtuseness of intellect, and that frequently a mind originally quick and vigorous, becomes sluggish and imbecile, need not be told to an assembly of physicians who have had the common opportunities of observing the effects of intemperance.

The stomach and liver of drunkards are generally found to be disordered; the stomach frequently contracted, and the liver much harder than natural, exhibiting an unnatural colour both upon its surface, and throughout its interior texture. This, perhaps, is what might be expected. The stomach receives the liquor, in the most concentrated and active form, in which it is taken into the body.—From the stomach and the alimentary canal below, most, if not all of it, is probably carried through the liver in a state less dilute than when distributed among the remaining organs of the body. The texture of the liver too, which consists merely of vessels and nerves with enough cellular bembrane to hold them together, may perhaps serve to show why it is more obviously affected than the alimentary canal, inasmuch as this canal has a distinct, and in some places, a thick muscular coat, independently of its vessels."

We hope the Professor—in whom we are happy to recognize a former class mate as well as a present collaborator—will continue his able exertions in the cause of *Temperance.*

